# Cumulative Weighted-Average Acuity Level: A Novel Clinician-Level Metric to Detect Emergency Department Surge Conditions in Real Time

**DOI:** 10.5811/westjem.66241

**Published:** 2026-10-13

**Authors:** Kenneth Berry

**Affiliations:** Lewis County General Hospital, Department of Emergency Medicine, Lowville, New York

## Abstract

**Introduction:**

Emergency department (ED) surge events, defined as the episodic clustering of high-acuity patients that transiently overwhelms available clinician capacity, are associated with delays in care and potential patient harm. Existing crowding metrics do not quantify real-time clinician-level workload. This paper describes the development and preliminary feasibility evaluation of a novel acuity-weighted metric—the cumulative weighted-average acuity level (CWAAL)—to detect physician-level surge conditions in real time.

**Methods:**

This multiphase observational feasibility study was conducted at two rural community EDs. The CWAAL is defined as the sum of weighted acuity scores for all active patients under a single clinician’s management: it incorporates a feasibility threshold derived by combinatorial enumeration. The primary outcome was door-to-doctor time (DDT); secondary outcomes included length of stay (LOS) and CWAAL distribution. The DDT comparisons at Hospital B (*n* = 4 surge events per condition) used the Mann-Whitney *U* test. All data were obtained from the practice of a single physician.

**Results:**

We evaluated a single emergency physician across 354 patients on 10 study dates at two rural community EDs, tracking 11 surge events and one proof of concept. A CWAAL feasibility threshold of ≤ 12 (±1)—equivalent to four simultaneous Level 3 patients (~120 minute demand within a 60-minute window)—emerged consistently across both sites. At Hospital B, surge-period DDTs were substantially lower with advanced practice clinician (APC) coverage than without (median, 31.5 minutes [interquartile range {IQR}, 24.7–38.0] vs 107.7 minutes [IQR, 85.7–133.5]); Mann-Whitney *U* = 0, *P* = .03, 2-tailed exact; rank-biserial *r* = 1.0), with complete distributional separation across eight surge events. All APC-supported events occurred at CWAAL ≤ 12; all unsupported events at CWAAL > 12. A proof-of-concept application on May 28, 2024, demonstrated operational feasibility; supplemental staffing reduced the physician’s weighted acuity burden by 31 points and normalized DDT and LOS.

**Conclusion:**

In this development cohort, the physician reached functional care capacity at cumulative weighted average acuity level = 12 (four simultaneous Level 3 patients; ~120 minutes demand within a 60-minute physician-hour). Complete separation of APC-supported and unsupported outcomes across eight events supports this novel acuity-weighted metric as a meaningful operational signal. Formal prospective validation across independent datasets is required before generalizing.

## INTRODUCTION

Emergency department (ED) crowding is a well-established threat to patient safety, associated with increased mortality, delays in care, and decreased quality of outcomes.^1^–^3^ Validated department-level crowding metrics—including the National ED Overcrowding Score (NEDOCS) and the Real-time Emergency Analysis of Demand Indicators (READI)—assess aggregate census, admission holds, and resource use.^4^ These tools are insensitive to a distinct and clinically important phenomenon: the episodic clustering of high-acuity patients that transiently overwhelms the capacity of a single available clinician, even when overall department census appears manageable.

In rural and community EDs operating under single-physician coverage models, this scenario is both common and dangerous. A physician managing three simultaneous Level 2 patients faces a fundamentally different operational reality than one managing the same census of lower-acuity patients, yet no existing metric captures this distinction in real time. Traditional downstream metrics such as door-to-doctor time (DDT) reflect surge impact only after care has already been compromised.

The conceptual foundation of this paper rests on the input-throughput-output model of ED crowding described by Asplin et al,^5^ which frames ED dysfunction as the product of mismatches between patient input rates, care throughput capacity, and output constraints. Queuing theory further suggests that when patient arrival rates transiently exceed service capacity, wait times increase nonlinearly and disproportionately to the degree of overload.^6^ A clinician-level acuity-weighted metric operationalizes this capacity constraint at the individual physician level.

This paper describes the development and preliminary feasibility evaluation of the cumulative weighted-average acuity level (CWAAL), a novel metric for real-time clinician-level surge detection. We report a multiphase observational study at two rural community EDs encompassing retrospective surge identification, analytical threshold derivation, and a prospective proof-of-concept application, intended to support formal prospective validation.

## METHODS

This study followed established methodological standards for medical record review in emergency medicine as described by Worster et al., including predefined variables, explicit outcome measures, and structured data abstraction.^7^ The SQUIRE 2.0 reporting checklist for quality improvement studies was followed where applicable. Data were obtained from the clinical practice of a single emergency physician at each study site, who is also the corresponding author of this paper. This relationship constitutes a potential source of observer and ascertainment bias, which is explicitly acknowledged in the Limitations section. Independent multiphysician replication remains the most important next step.

### Study Design and Setting

This feasibility study was conducted in three sequential phases across two rural community EDs: (1) Hospital A—a community ED with approximately 22,000–24,000 annual visits, 18–22 beds, and Meditech electronic health record (EHR); and (2) Hospital B—a smaller rural ED with approximately 11,000–12,000 annual visits, 10–14 beds, and Meditech EHR. Neither facility employed physician scribes during the study period. This study was determined to constitute quality improvement activity and to be exempt from formal review pursuant to 45 CFR 46.104(d). In compliance with the Health Insurance Portability and Accountability Act, we de-identified all data prior to analysis. De-identified data from Hospital A were provided by the hospital director of medical records with institutional authorization.

**Population Health Research Capsule**

What do we already know about this issue?

*Emergency department (ED) crowding harms patients, but no metric quantifies real-time workload for an individual clinician.*

What was the research question?

*Can a clinician-level acuity-weighted metric detect physician-level surge conditions in real time?*

What was the major finding of the study?

*Advanced practice clinician (APC)-supported surges had lower door-to-doctor time (DDT) than unsupported surges (31.5 vs 107.7 min; P = .03; r = 1.0).*

How does this improve population health?

*This novel acuity-weighted metric could enable earlier staffing response to surges, reducing delays and improving rural ED capacity.*

### Metric Development

The CWAAL metric was developed through a sequential, three-phase process. In Phase 1, retrospective review of Hospital A surge events identified a recurring pattern: clustered arrivals of high-acuity patients within discrete time windows consistently produced marked DDT elevations despite adequate overall staffing, motivating development of a clinician-level workload measure. We adapted a five-level acuity classification already in clinical use for triage by inverting and weighting the levels to reflect approximate physician time demands per encounter: Level 1 (Critical) = weight 5 (50 min); Level 2 (Emergent) = 4 (40 min); Level 3 (Urgent) = 3 (30 min); Level 4 (Nonurgent) = 2 (20 min); Level 5 (Minor) = 1 (10 min). These time estimates are heuristic, derived from clinical experience, and should be considered working approximations pending formal time-motion validation. Levels 1/2 and 4/5 were combined for analytical tractability given small cell sizes at the extremes. We then defined CWAAL as the sum of weighted acuity scores for all patients under a clinician’s active management within a defined time window. (“Average” in the acronym reflects the acuity-weighting concept rather than arithmetic averaging; CWAAL is a cumulative sum, not a mean). Patients partially managed prior to the surge interval were assigned a 0.5 × reduced weight to account for completed work.

In Phase 2, the Hospital B dataset provided natural comparisons across varying staffing configurations. To derive a formal feasibility threshold, all possible patient acuity combinations for Levels 2, 3, and 4 were systematically enumerated: 81 four-patient combinations (categories 8–16) for Hospital A and 27 three-patient combinations (categories 6–12) for Hospital B. Each combination was evaluated against single-physician capacity within a one-hour window. The critical inflection point emerged at CWAAL = 12: combinations at ≤ 11 were consistently feasible within 60 minutes while combinations at ≥ 12 exceeded single-physician capacity (eg, four simultaneous Level 3 patients (4 × 3 = 12) represent approximately 120 minutes of care demand, exceeding the 60-minute window). Full combinatorial tables appear in Supplementary Appendix A, which also contains a generalized mathematical formalization of the CWAAL framework applicable to multizone, multiclinician EDs.

Importantly, this threshold was derived from and evaluated against the same dataset—a recognized limitation of exploratory metric development. The CWAAL = 12 threshold represents a hypothesis generated from preliminary data, rather than a validated cut-point. The 0.8 physician-equivalent weighting assigned to APC clinicians (yielding a physician-APC team threshold of ≤ 18) reflects retrospective modeling of the DDT data and is an empirical estimate requiring prospective calibration.

### Patient Acuity Framework

Table 1 summarizes the five-level acuity classification and associated weighted scores. The Approximate Time Requirement column represents the heuristic time demand per encounter, derived using a 10-min base unit (weight × 10 min), pending formal time-motion validation.

### Surge Event Definition

We defined a surge event operationally as the sudden arrival of more than three patients of acuity level 3 or higher within any 60-minute period per available emergency physician. This definition was developed pragmatically from observed clustering patterns and is an operational working definition; its sensitivity and specificity relative to alternative definitions have not been formally evaluated.

#### Phase 1: Hospital A Data (January–June 2019)

The DDT data from Hospital A were retrieved and de-identified by the hospital director of medical records. Records from a single emergency physician were analyzed chronologically. Surge events were identified on February 2, March 11, and May 20, 2019; June 2, 2019—a nonsurge day with comparable volume but lower acuity and adequate staffing—served as the descriptive control. All four shifts operated under a staffing formula of one emergency physician (7 am to 7 pm), one advanced practice clinician (APC) (9 am to 11 pm), and a second APC beginning at 2 pm or 4 pm. Phase 1 data are reported descriptively.

#### Phase 2: Hospital B Data (July–November 2021)

A secondary dataset was obtained from Hospital B covering the same physician across multiple surge and control dates. Hospital B staffing included a single emergency physician (7 am to 7 pm) and one APC daily (9 am to 8 pm). Key comparisons were drawn between surge dates with APC coverage (August 1, October 3, 2021), without APC coverage (September 15, September 29, 2021), and a within-shift control date (August 25, 2021) on which both an APC-supported and an unsupported surge occurred.

### Statistical Analysis

We used nonparametric methods throughout given small sample sizes. The DDT comparisons between APC-supported and unsupported surge conditions used the Mann-Whitney *U* test. Two complementary analyses were conducted: (1) a surge-level analysis (Hospital B only; *n* = 4 with APC, *n* = 4 without APC); and (2) a patient-level analysis using individual DDTs from the EHR throughput reports (*n* = 14 with APC, *n* = 11 without). The patient-level analysis carries a nonindependence caveat—patients within a shared surge share common conditions—and is reported as corroborating evidence. Effect size was quantified using the rank-biserial correlation coefficient (*r*), where |*r*| = 1.0 indicates complete separation and |*r*| ≥ .5 is conventionally large. With *n* = 4 per group, *P* (two-tailed exact) represents the minimum achievable *P* value for this test; the clinical magnitude of the effect is therefore the primary evidentiary basis. Data are reported as median [IQR]. We conducted all analyses in Python (scipy.stats, version 1.13). No inferential statistics were applied to Phase 1 or Phase 3 data, which are reported descriptively.

#### Phase 3: Prospective Proof-of-Concept Application (May 28, 2024—Hospital B)

The Tuesday following Memorial Day 2024 was selected prospectively as a high-probability surge day based on historical census patterns. A precoordinated surge response bundle was implemented: advance staff briefing, preactivation of additional information technology workstations, float nursing augmentation, commitment of a supplemental physician to respond by noon if CWAAL-defined surge criteria were met, prenotification of hospital administration and emergency medical services (EMS), and real-time CWAAL calculation. The surge response bundle was precoordinated rather than triggered reactively by CWAAL; CWAAL therefore functioned as a monitoring and communication tool within an already activated framework, not as an independent trigger. This phase demonstrates operational feasibility of real-time CWAAL calculation, rather than whether CWAAL-triggered responses improve outcomes versus unmanaged surge. We used a pre-post comparative design, matching the surge period (approximately 10 am to 2 pm) against the non-surge period (after 7 pm). All Phase 3 analysis was descriptive.

## RESULTS

### Patient Characteristics

Across both study sites, a total of 354 patients were evaluated over 10 shifts by a single emergency physician (140 at Hospital A across 4 shifts, January–June 2019; 214 at Hospital B across 6 shifts, July 2021–May 2024). Patient characteristics by study date are summarized in Table 2. Across all Hospital B surge dates, total shift volume ranged from 32 to 36 patients per 24-hour period. Surge-window patients were predominantly Acuity Level 2-3 (100% across all dates). Age ranged from infant to 93 years; sex distribution was mixed. Dispositions included home discharge, admission, transfer, and—particularly during unsupported surges—left-without-being-seen and against-medical-advice departure, consistent with the throughput delays documented below. Hospital A (Phase 1) individual patient records (age, sex, LOS) were not recoverable after relocation; however, aggregate shift data including patient volumes, acuity distributions, peak DDT values, and CWAAL threshold confirmation were subsequently recovered from structured data abstraction worksheets (Exhibits C1–C4; Supplementary Appendix B) and are presented in Table 2.

#### Phase 1: Hospital A Surge Events

Across three Hospital A surge events (February 2, March 11, and May 20, 2019), clustered arrivals of high-acuity patients (≥ 7 patients of Level 2–3 within approximately 2–3 hours) were consistently associated with DDT increases ranging from 82 to 162 minutes. Shift volumes of approximately 30–40 patients per shift were identified from recovered data abstraction worksheets (Exhibits C1–C4; Supplementary Appendix B); individual patient age, sex, and LOS data were not recoverable. On February 2 and March 11, DDTs normalized with the scheduled arrival of the second APC; on May 20, normalization required call-in of an additional clinician, as scheduled staffing was insufficient given concurrent nursing throughput and bed availability constraints. This finding illustrates that clinician availability alone is insufficient when nursing and bed capacity are simultaneously constrained. The June 2, 2019, control date (30 patients: 0 Level 1, 2 Level 2, 16 Level 3, 12 Level 4, 0 Level 5) demonstrated DDTs within acceptable limits throughout, consistent with a well-staffed nonsurge shift.

#### Phase 2: Hospital B Surge Events

Table 3 summarizes surge metrics for all Hospital B dates. With APC coverage (August 1 Surges 1 and 2, October 3, August 25 Surge 1), average surge-period DDTs ranged from 18.7 to 43.5 minutes; all four APC-supported events had CWAAL ≤ 12. Without APC coverage (September 15, September 29 Surges 1 and 2, August 25 Surge 2), average surge-period DDTs ranged from 71.7 to 159 minutes—a 3- to 6-fold increase; all four unsupported events had CWAAL > 12.

Across all 8 Hospital B surge events, DDTs were lower with APC coverage (median, 31.5 min; IQR, 24.7–38.0 min), than without (median, 107.7 min; 107.7 min; IQR, 85.7–133.5 min; Mann-Whitney *U* = 0; P = .03, two-tailed exact; rank-biserial r = 1.0). Complete separation was observed within this limited dataset: every APC-supported surge DDT was lower than every unsupported surge DDT. At the individual patient level, the difference remained robust (Mann-Whitney *U* = 19.5; P < .001; rank-biserial r = 0.75; n = 14 with APC; n = 11 without), with a median DDT of 22 min versus 128 min. These patient-level metrics are reported as corroborating evidence given patient nonindependence within surge events.

Arrival-to-discharge times followed the same pattern: 147–196 minutes with APC versus 270–325 minutes without. The August 25 within-shift comparison is of particular note: Surge 1 (APC present, 3:34 pm to 4:24 pm) produced median DDT of 18.7 minutes; Surge 2 (APC absent, 7:01 pm to 8:01 pm) produced 71.7 minutes—a 3.8-fold difference on the same date, same facility, same physician, providing the most internally controlled evidence in this dataset for the independent contribution of APC staffing.

### Threshold Analysis

Retrospective application of the CWAAL model produced consistent results: CWAAL ≤ 12 (±1) corresponded to all feasible single-physician scenarios; 13–17 represented a margin zone; ≥ 18 (±2) corresponded to all scenarios with significantly elevated DDTs. With a physician-APC team, the feasibility threshold rose to ≤ 18 (±2). These thresholds are summarized in Table 4. They were derived from and evaluated on the same dataset and represent preliminary working thresholds, not validated cut-points.

#### Phase 3: Proof-of-Concept Application—May 28, 2024

The surge commenced at approximately 10 am with nine patients arriving by 10:59 am and four more by 11:59 am. The physician in charge began with baseline CWAAL of 3.5; six patients assumed from 9 am to 10:38 am yielded CWAAL of 20.5, well above the ≥ 18 alarm threshold. Including two additional high-acuity arrivals at 11:11 am and 11:47 am, the CWAAL of the physician in charge reached 27.5 over the three-hour surge window. Table 5 summarizes clinician contributions; precoordinated supplemental staffing arriving 12 pm to 2 pm provided 31 CWAAL units of assistance. The EMS diversion at 2 pm further decompressed census.

The DDTs for the first 7 patients ranged from 1 to 54 minutes; patients 8–14 experienced escalating DDTs peaking at 349 minutes before normalizing following supplemental staffing. The LOS mirrored this pattern: patients arriving 10:00 am to 10:59 am averaged 5 hours 23 minutes versus 2 hours 38 minutes to 2 hours 57 minutes for patients arriving after 1 pm. After 7 pm data confirmed normalization. Two inpatient admissions and two transfers occurred during the surge. The throughput improvement reflects the precoordinated response bundle as a whole—including nursing augmentation, physician backup, and EMS diversion—rather than CWAAL-triggered intervention alone; CWAAL served as the quantitative workload framework enabling coordination and documentation of surge severity.

## DISCUSSION

### Interpretation and Summary of Main Findings

This study presents the development and preliminary feasibility evaluation of CWAAL, a clinician-level, acuity-weighted tool for real-time ED surge detection, evaluated across 354 patients on 10 study dates at two rural community EDs. The central finding—that a CWAAL threshold of ≤ 12 (±1) emerged consistently across two rural community EDs and corresponded with observed complete separation of APC-supported and unsupported surge outcomes within this limited dataset—is consistent with the hypothesis that CWAAL captures a meaningful operational signal. These results are preliminary; they establish a foundation for formal prospective validation rather than providing definitive evidence of generalizability.

The most striking feature of the Phase 2 data is not statistical significance—which represents the minimum achievable *P* value for this sample size—but the clinical magnitude and completeness of the separation. A 3.4-fold median DDT difference and zero distributional overlap across eight heterogeneous surge events constitutes a robust preliminary signal. The within-shift August 25 comparison is particularly compelling: the same physician, facility, and date produced a 3.8-fold DDT difference based solely on APC presence during the surge window, providing the closest approximation to an internal control available in this dataset.

### Comparison to Previous Studies

Prior literature has demonstrated that team-based staffing models improve ED throughput efficiency.^8^ The CWAAL extends this finding by providing the first quantified, clinician-level estimate of the workload threshold at which a single physician’s DDT performance degrades during surge conditions in a rural community ED. The feasibility ceiling of ≤ 18 for a physician-APC team reflects the additive capacity conferred by APC support and may offer a specific operational target for staffing calculations, pending independent validation.

Existing ED crowding metrics—including NEDOCS and READI—assess department-level crowding using aggregate metrics such as total census, admission holds, and ventilator patients.^4^ The CWAAL is complementary but operationally distinct: a department with 12 patients and one physician managing four simultaneous Level 2 patients may appear unremarkable on NEDOCS, while that physician’s CWAAL is well above the alarm threshold. The CWAAL is not a replacement for these tools but operates at a different level of resolution—the individual clinician rather than the department.

### Strengths

The primary strength of this novel metric is a clinically intuitive operational signal that demonstrated consistent behavior across two independent rural community EDs, multiple staffing configurations, varying patient volumes, and four calendar months of heterogeneous clinical conditions. The within-shift August 25 comparison provides an internal control for clinician, facility, and date factors unavailable in most observational designs. Phase 3 demonstrated that real-time CWAAL calculation is operationally feasible using existing EHR data with no additional technology infrastructure. This study fills a specific gap: No prior tool has attempted to quantify individual clinician acuity burden in real time as a prospective surge detection mechanism in rural emergency medicine.

### Clinical Implications

The May 20, 2019, Hospital A surge illustrates a critical boundary condition: Prolonged DDTs occurred despite concurrent physician and APC availability because nursing throughput and bed capacity were simultaneously constrained. The CWAAL measures physician acuity burden only; it does not incorporate nursing staffing, bed availability, ancillary response times, or output constraints, all of which independently contribute to surge severity. Surge response protocols built on CWAAL should treat physician workload as one component of a multidimensional operational assessment, rather than a sufficient summary measure.

In practice, CWAAL could be automatically calculated from triage data in standard EHR systems and displayed within the ED tracking board, enabling tiered surge response activation without physician distraction. The conceptual architecture—watch at CWAAL ≥10, alert at CWAAL ≥12, alarm at CWAAL ≥18 — maps naturally onto existing hospital incident command infrastructure.

### Research Implications and Future Directions

This study establishes CWAAL as a quantitative, clinician-level framework with demonstrated preliminary feasibility. Priority next steps are as follows: (1) formal prospective validation on independent datasets across multiple physicians, sites, and staffing configurations with preregistered hypotheses; (2) time-motion studies to calibrate acuity-weight assignments empirically; (3) EHR integration pilot studies with automated CWAAL calculation and tiered alert protocols; (4) outcomes studies linking CWAAL-triggered interventions to measurable patient safety endpoints; and (5) formal cost-effectiveness analysis. Regulatory and institutional policy applications should follow, not precede, completion of these steps.

If prospectively validated, CWAAL applied retrospectively to EHR-derived DDT and acuity data could provide an objective characterization of clinician workload patterns over time, informing institutional staffing decisions and quality improvement programs. Understaffing of EDs has been documented as a contributor to crowding-related patient harm and clinician burnout.^1^–^3^,^9^,^10^ These regulatory applications are speculative at this stage and require formal validation before any institutional or regulatory use.

## LIMITATIONS

This study has several limitations. First and most fundamentally, all data were obtained from the clinical practice of a single physician who is also the study’s corresponding author. Every subjective judgment in the methodology—surge event identification, acuity-weight assignments, feasibility determinations, partial-weight discounting—was made by this individual, representing a significant potential source of observer, confirmation, and ascertainment bias. Independent replication by other physicians at other sites is the most important next step.

Independent adjudication of surge events was not performed. Surge identification and threshold exploration were conducted using prospectively recorded workload data from a single clinician. Future studies should evaluate inter-rater reliability and external validation across multiple clinicians and institutions. Additionally, the CWAAL threshold of ≤ 12 was derived from and evaluated on the same dataset. This circular derivation is a recognized limitation of exploratory metric development; the threshold’s apparent predictive validity may partly reflect overfitting to the development data. Prospective evaluation on independent datasets is required before the threshold can be considered validated.

We did not perform multivariable analysis given sample sizes of *n* = 4 surge events per condition; such analyses would be statistically underpowered. The between-condition comparisons cannot control for differences in patient population characteristics, time of day, or seasonal variation. The within-shift August 25 comparison provides partial internal control but does not eliminate all confounding.

The Phase 3 proof-of-concept application involved precoordinated staffing and heightened situational awareness. It does not constitute an independent test of the CWAAL threshold and cannot isolate CWAAL’s contribution from other response bundle components. Multiple prospective surge days across varied settings with independent adjudication will be required to establish threshold robustness.

Acuity-weight assignments are derived from clinical experience rather than formal time-motion studies, and CWAAL measures physician acuity burden only—not nursing capacity, bed availability, ancillary response times, or output constraints, all of which independently contribute to surge severity. Sixth, the conceptual framework may not generalize across different patient populations or time periods without recalibration; both study sites share characteristics—modest annual volume, single-physician coverage, limited bed capacity, no scribes—that may not apply to academic or high-volume urban EDs. Finally, downstream patient safety outcomes—including adverse events, patient satisfaction,*11* and formal quality metrics*—were not measured* and represent the ultimate measure of this framework’s clinical value.

## CONCLUSION

Surge events in the emergency department are quantifiable at the individual clinician level using the cumulative weighted-average acuity level metric. Preliminary data from two rural community EDs demonstrate a striking and consistent association between CWAAL threshold exceedance and door-to-doctor degradation across eight heterogeneous surge events, with observed complete separation of advance practice clinician-supported and unsupported surge outcomes within this limited dataset. A prospective proof-of-concept application confirmed operational feasibility of real-time CWAAL calculation using existing EHR data.

These findings are explicitly preliminary and hypothesis-generating. The proposed CWAAL threshold of ≤ 12 (±1) for a single emergency physician and ≤ 18 (±2) for a physician-APC team represents an analytically consistent working hypothesis from a limited single-physician dataset, not a validated universal standard. Formal prospective validation across independent data, multiple physicians, and diverse ED settings is required before these thresholds can be adopted operationally. Subject to that validation, CWAAL has potential as a practical early-warning tool for surge detection complementing existing department-level crowding metrics—with longer-term potential for staffing quality improvement contingent on the validation work that must come first.

## Supplementary Information





## Figures and Tables

**Table 1:** Patient acuity classification and associated weighting used to calculate a novel acuity-weighted metric to detect physician-level surge conditions in real time in a study of rural emergency departments.

Acuity Level	Clinical status	Weighted score	Approx. time requirement (min)	Time basis
1 (Critical)	Immediate threat to life and/or limb	5	~50	Weight × 10 min (heuristic)
2 (Emergent)	Impending threat to life and/or limb	4	40	Weight × 10 min (heuristic)
3 (Urgent)	Urgent illness requiring immediate care	3	30	Weight × 10 min (heuristic)
4 (Non-urgent)	Medical need without immediate life threat	2	20	Weight × 10 min (heuristic)
5 (Minor)	Could be treated elsewhere at another time	1	10	Weight × 10 min (heuristic)

Weight assignments reflect estimated time demands derived from clinical experience and should be considered heuristic pending formal time-motion validation. Levels 1/2 and 4/5 were combined for analytical tractability.

**Table 2:** Patient characteristics by study date in a multiphase observational feasibility study evaluating a novel acuity-weighted metric to detect physician-level surge conditions in real time in a study of rural emergency departments.

Date/surge	APC present	Shift total pts	Shift avg LOS (min)	Surge pts (N)	Surge age range	Surge sex (M:F)	Surge Acuity Levels	Surge dispositions	Surge Avg DDT (min)	CWAAL category
HOSPITAL A — PHASE 1 RETROSPECTIVE DATA (January–June 2019)										
2/2/19 Surge	Yes (2nd APC arrived scheduled)	~40*	N/A†	~10–15 (surge window)	N/A†	N/A†	L1:0|L2:2|L3:17|L4:17|L5:4 (shift); surge: L2–3 clustered	DDTs normalized w/ scheduled 2nd APC arrival	Peak DDT 162 min; pre-surge 8–37 min	> 12 confirmed (see Methods)
3/11/19 Surge	Yes (2nd APC arrived scheduled)	36	N/A†	~16 (surge window)	N/A†	N/A†	L1:0|L2:5|L3:22|L4:9|L5:0 (shift); surge: L2–3 clustered	DDTs normalized w/ scheduled 2nd APC arrival	Peak DDT ~102 min; CWAAL 15→16–>19	> 12 confirmed (CWAAL 15–19)
5/20/19 Surge	Yes (additional clinician called in)	~37*	N/A†	~15 (surge window)	N/A†	N/A†	L1:0|L2:6|L3:13|L4:14|L5: ~4 (shift); surge: L2–3 + nursing constraint	Req. extra clinician call-in; nursing & bed capacity limited	Peak DDT 159 min despite MD+APC (nursing/bed constraint)	> 12 confirmed; nursing constraint
6/2/19 Control (Non-surge)	Yes (standard formula)	30	N/A†	30 (full shift)	N/A†	N/A†	0 L1 | 2 L2 16 L3 | 12 L4 0 L5	All home/ acceptable; 1 registration delay only	Within normal limits	N/A (non-surge)
WITH APC COVERAGE (Hospital B, 2021)										
8/1/21 S1	Yes	36	245	5	20–82	3M:4F	Level 2: 2 Level 3: 3	3 admitted 2 home	36.2	≤ 12
8/1/21 S2*	Yes	36	245	3*	20–82	0M:3F	Level 2: 2 Level 3: 1	1 admitted 1 home 1 unknown	43.5*	≤ 12
10/3/21	Yes	35	179	3	67–93	0M:3F	Level 3: 3	1 home 1 VA transfer 1 NH return	26.7	≤ 12
8/25/21 S1+	Yes	32	202	4	15–43	3M:1F	Level 2: 1 Level 3: 3	1 transfer 3 home	18.7	≤ 12
WITHOUT APC COVERAGE (Hospital B, 2021)										
8/25/21 S2+	No	32	202	3	Infant –28	2M:1F	Level 3: 3	2 home 1 unknown	71.7	> 12
9/15/21	No	34	309	3	37–81	2M:1F	Level 2: 1 Level 3: 2	1 transfer 1 AMA 1 home	125.0	> 12
9/29/21 S1	No	35	325	3	26–74	1M:2F	Level 3: 3	1 LWBS 1 AMA 1 home	159.0	> 12
9/29/21 S2	No	35	325	3	2y–70	2M:1F	Level 3: 3	1 home 1 elopement 1 admitted	90.3	> 12
PHASE 3: PROOF-OF-CONCEPT APPLICATION (Hospital B, 5/28/24)										
5/28/24 Surge	Yes (arr. noon)	42	271	13 surge window	5–76 (n = 20)	~12M:9F (n = 21)	L2: ~5 L3: ~6 L4: ~2 (surge window)	2 admitted 2 transfer 1 AMA 1 LWBS Remainder home	Peak 349; normalized post-response	Peak CWAAL 27.5

† Hospital A (Phase 1) individual patient records (age, sex, LOS) were not recoverable after relocation. Aggregate shift data (patient volumes, acuity distributions, peak DDT, and CWAAL threshold confirmation) were subsequently recovered from structured data abstraction worksheets (Exhibits C1–C4) and are reported here.

* Shift totals are approximate counts from recovered worksheets. Age, sex, and shift LOS remain unavailable. CWAAL was not calculated prospectively during Phase 1; values shown reflect retrospective confirmation of threshold exceedance (see Methods). Control date DDT excludes one outlier (186 min) attributable to a documented registration delay. Shift volume: 30–40 patients across Phase 1 dates (Exhibits C1–C4). CWAAL was not calculated prospectively during Phase 1; threshold derivation used Hospital A data retrospectively to establish combinatorial feasibility categories (see Methods). Staffing formula: 1 emergency physician (7 am–7 pm) + 1 APC (9 am–9 pm) + 2nd APC beginning at 2 pm or 4 pm on all four dates.

* 8/1/21 Surge 2: DDT average based on 2 patients with complete data; sex for remaining patient indeterminate.

+ 8/25/21: Surge 1 (with APC) and Surge 2 (without APC) occurred same date, same physician, same facility; shift totals and LOS therefore identical.

5/28/24 age/sex from registration clerk log (first 20–21 of 38 patients). Hospital A age/sex/LOS not recoverable; aggregate shift data from Exhibits C1–C4.

*AMA*, against medical advice; *APC*, advanced practice clinician; *CWAAL*, cumulative weighted-average acuity level; *DDT*, door-to-doctor time; *LOS*, length of stay; *LWBS*, left without being seen; *NH*, nursing home; *VA*, Veterans Affairs.

**Table 3:** Door-to-doctor times during emergency department surge events with and without advanced practice clinician support in a study of rural emergency departments evaluating the cumulative weighted-average acuity level metric.

Date	Surge window	APC present	Mean DDT (min)	Mean AtD (min)	Pts in ED	CWAAL category	Note
WITH APC COVERAGE							
8/1 S1	5:02 pm – 5:49 pm	Yes	36.2	298.2	9	≤ 12	
8/1 S2	10:11 pm – 11:12 pm	Yes	43.5	--	10	≤ 12	^*^2 pts w/ complete data
10/3	1:36 pm – 2:43 pm	Yes	26.7	350.3	8	≤ 12	
8/25 S1	13:34 pm – 4:24 pm	Yes	18.7	282.0	9	≤ 12	+Within-shift control
WITHOUT APC COVERAGE							
9/15	3:49 pm – 4:56 pm	No	125.0	662.3	16	> 12	
9/29 S1	1:06 pm – 2:14 pm	No	159.0	270.7	15	> 12	
9/29 S2	5:18 pm – 6:03 pm	No	90.3	241.7	12	> 12	
8/25 S2	7:01 pm – 8:01 pm	No	71.7	162.3	13	> 12	+Within-shift control

Median DDT: 31.5 min (with APC) vs 107.7 min (without APC); Mann-Whitney *U* = 0, *P* = .03, 2-tailed exact; rank-biserial *r* = 1.0.

+ 8/25/21 Surge 1 (with APC) and Surge 2 (without APC) occurred same date, same physician, same facility — providing within-shift internal control for clinician, facility, and patient population factors.

*APC*, advanced practice clinician; *AtD*, arrival-to-discharge time; *CWAAL*, cumulative weighted-average acuity level; *DDT*, door-to-doctor time.

**Table 4:** Proposed cumulative weighted-average acuity level thresholds for clinician feasibility and surge response in a study of rural emergency departments.

CWAAL	Feasibility assessment	Clinical implication
≤ 12 (±1)	Feasible	A single emergency physician can manage patient load safely
13–17	Marginal / Alert Zone	Surge alert warranted; additional clinician support (eg, APC or physician) should be considered
≥ 18 (±2)	Not Feasible	Surge alarm; immediate supplemental staffing required
≤ 18 (with APC)	Feasible (team)	Combined physician and APC team can manage surge load

These thresholds were derived from and evaluated on the same retrospective dataset and should not be interpreted as externally validated cut-points.

*APC*, advanced practice clinician; *CWAAL*, cumulative weighted-average acuity level.

**Table 5:** Clinician contributions during a prospective proof-of-concept emergency department surge event on May 28, 2024, in a rural community emergency department study evaluating the cumulative weighted-average acuity level metric.

Clinician	Role during surge	Patients seen	CWAAL units	Arrival time
Physician in Charge	Primary emergency physician	8 (full); 4 assigned to APC	27.5	On duty 7:00 am
APC	Advanced practice clinician	4 (surge); 3 (post-surge)	11.5	Noon
Additional emergency physician #1	Surge responder	1	3.0	1:00 pm
Additional emergency physician #2	Replacement clinician	2 (including 1 partial)	4.5 (1.5 shared)	1:50 pm
Surgeon	Surge responder	1	3.0	~1:00 pm
Total assistance to physician in charge			31.0	

*APC*, advanced practice clinician; *CWAAL*, cumulative weighted-average acuity level.

## References

[1] Pines JM, Pilgrim RL, Schneider SM (2011). Practical implications of implementing emergency department crowding interventions: a systematic review. Ann Emerg Med.

[2] Bernstein SL, Aronsky D, Duseja R (2009). The effect of emergency department crowding on clinically oriented outcomes. Acad Emerg Med.

[3] Hoot NR, Aronsky D (2008). Systematic review of emergency department crowding: Causes, effects, and solutions. Ann Emerg Med.

[4] Weiss SJ, Derlet R, Arndahl J (2004). Estimating the degree of emergency department overcrowding in academic medical centers: Results of the National ED Overcrowding Study (NEDOCS). Acad Emerg Med.

[5] Asplin BR, Magid DJ, Rhodes KV (2003). A conceptual model of emergency department crowding. Ann Emerg Med.

[6] Green LV, Hall RW (2006). Queueing analysis in healthcare. Patient Flow: Reducing Delay in Healthcare Delivery.

[7] Worster A, Bledsoe RD, Cleve P (2005). Reassessing the methods of medical record review studies in emergency medicine research. Ann Emerg Med.

[8] Rowe BH, Guo X, Villa-Roel C (2011). The role of triage liaison physicians on mitigating crowding in emergency departments: a systematic review. Acad Emerg Med.

[9] Kellermann AL (2006). Crisis in the emergency department. N Engl J Med.

[10] Pines JM, Garson C, Baxt WG (2007). ED crowding is associated with variable perceptions of care compromise. Acad Emerg Med.

[11] Welch SJ (2010). Twenty years of patient satisfaction research applied to the emergency department: a qualitative review. Am J Med Qual.

