# Supplementary material for: Cumulative Weighted-Average Acuity Level: A Novel Clinician-Level Metric to Detect Emergency Department Surge Conditions in Real Time
Source: West J Emerg Med. 2026 Oct 13;27(6):1516–24. doi: 10.5811/westjem.66241 (PMC13761938; doi:10.5811/westjem.66241)
Supplement: Supplementary file 1 [file wjem-27-1516-s002.pdf]

Supplementary Appendix B: Exhibits C1-C4

(Recovered selected Hospital A Data Worksheets—Phase 1

Retrospective Analysis (January—June 2019))

Exhibit C1

(Selected Data For Analysis Based on Surge Event Criteria)

1. Data of February 2, 2019

Feb 2, 2019

① = 0 ② = 2 ③ = 17 ④ = 17 ⑤ = 4

[illegible]	1/31/19 11:09	January	11	4	
1583	2/2/19 8:30	February	15	3	
417	2/2/19 8:50	February	13	4	No ML arrival
~~668~~	~~2/2/19 9:12~~	~~February~~	~~21~~	~~3~~	
473	2/2/19 10:28	February	14	5	
573	2/2/19 10:47	February	17	5	
574	2/2/19 11:09	February	8	4	No ML arrival
1584	2/2/19 11:12	February	15	2	
669	2/2/19 11:35	February	6	4	
474	2/2/19 11:43	February	8	4	
418	2/2/19 11:46	February	20	4	
1585	2/2/19 11:52	February	33	3	
1426	2/2/19 12:06	February	15	4	
575	2/2/19 12:13	February	11	3	
~~419~~	~~2/2/19 12:14~~	~~February~~	~~13~~	~~4~~	
576	2/2/19 12:23	February	37	3	
1587	2/2/19 12:48	February	19	3	
420	2/2/19 12:58	February	20	4	
475	2/2/19 13:00	February	18	3	
476	2/2/19 13:11	February	37	3	
421	2/2/19 13:12	February	92	4	20
477	2/2/19 13:22	February	12	2	+1
1588	2/2/19 13:24	February	91	4	
577	2/2/19 13:34	February	82	4	
478	2/2/19 13:42	February	96	4	
422	2/2/19 13:47	February	24	3	
1427	2/2/19 13:47	February	24	3	
423	2/2/19 13:57	February	* ⇒ 162	3	→ ML Arrival (by memory)
479	2/2/19 14:02	February	110	4	
1428	2/2/19 14:10	February	52	3	ML — 14:15 actual
1589	2/2/19 14:23	February	57	3	30
670	2/2/19 14:47	February	35	3	
578	2/2/19 15:19	February	14	3	
1586	2/2/19 15:39	February	48	3	
579	2/2/19 16:22	February	17	4	
1590	2/2/19 16:37	February	21	5	35
424	2/2/19 17:01	February	39	5	38
580	2/2/19 17:32	February	10	4	
581	2/2/19 17:38	February	24	4	
1591	2/2/19 17:39	February	13	4	
480	2/2/19 17:41	February	23	3	40

Feb 2 Shift

① = 0 ② = 2 ③ = 17 ④ = 17 ⑤ = 4

[illegible]	[illegible]	January		11	4			
1583	2/2/19 8:30	February		15	3			
417	2/2/19 8:50	February		13	4			No ML arrival
668	2/2/19 9:12	February		21	3			
473	2/2/19 10:28	February		14	5			
573	2/2/19 10:47	February		17	5			No ML arrival.
574	2/2/19 11:09	February	11:17	8	4			
1584	2/2/19 11:12	February	11:27	15	2			
669	2/2/19 11:35	February	11:41	6	4			
474	2/2/19 11:43	February	11:57	8	4			
418	2/2/19 11:46	February	12:06	20	4			
1585	2/2/19 11:52	February	12:25	33	3			
1426	2/2/19 12:06	February	12:31	15	4			
575	2/2/19 12:13	February	12:24	11	3			
419	2/2/19 12:14	February	12:37	13	4			
576	2/2/19 12:23	February	13:00	37	3			
1587	2/2/19 12:48	February	13:07	19	3	[illegible]		
420	2/2/19 12:58	February	13:18	20	4			
475	2/2/19 13:00	February	13:18	18	3			
476	2/2/19 13:11	February	13:48	37	3			
421	2/2/19 13:12	February	14:44	92	4	[illegible]	15	20
477	2/2/19 13:22	February	13:34	12	2	[illegible]	15	
1588	2/2/19 13:24	February	14:55	91	4	ML		
577	2/2/19 13:34	February	14:46	82	4	ML		
478	2/2/19 13:42	February	14:48 ML	96	4	ML		
422	2/2/19 13:47	February	14:11	24	3	MD		
1427	2/2/19 13:47	February	14:11	24	3	MD		1 (only)
423	2/2/19 13:57	February	16:37	162	3	ML		ML arrival
479	2/2/19 14:02	February	15:53	110	4	ML		(by [illegible])
1428	2/2/19 14:10	February	15:02	52	3	MD		ML — 14:15
1589	2/2/19 14:23	February	15:20	57	3	MD		30 actual
670	2/2/19 14:47	February	15:22	35	3	MD		
578	2/2/19 15:19	February		14	3			
1586	2/2/19 15:39	February		48	3			
579	2/2/19 16:22	February		17	4			
1590	2/2/19 16:37	February		21	5			35
424	2/2/19 17:01	February		39	5			38
580	2/2/19 17:32	February		10	4			
581	2/2/19 17:38	February		24	4			
1591	2/2/19 17:39	February		13	4			
480	2/2/19 17:41	February		23	3			40

Feb 2
surge

Pts 1 → 5

	# wulf	
11:17	4 → 2	
11:27	2 → 4	10 ut pts / 24 min
11:41	4 → 2	
11:51	4 → 2	limited S. Akers
12:06	4 → 2	help arrived
49 min (~50 min)	18 / 12	

3 pts / 24 min (1/2 hr)

2 pts / 15 min

1st hr

DTs =

64 min

[15 minutes (12:06 → 12:21)]

pts 6 → 10

12:25	3 – 3
12:21	4 – 2
12:24	3 – 3
12:27	4 – 2
13:00	3 – 3
39 min	17 / 13

5 pts / 39 min

2nd hr

DTs =

[7 minutes (1300 – 1307)]

64 + 39 + 7 = 110 min

pts 11 → 15

13:07	3 – 3
13:18	3 – 3
13:19	3 – 3
13:48	4 – 2
13:34	2 – 4
27 min	/ 15

5 pts / 27 min

DTs =

15 PATIENTS / 137 minutes

9.13 Pt / HR.

Exhibit C2

(Selected Data For Analysis Based on Surge Event Criteria)

2. Data of March 11, 2019

march 11
2019

582	3/11/19 5:15	March		6	3		
481	3/11/19 8:01	March		10	3		
425	3/11/19 8:11	March		14	3		
583	3/11/19 8:45	March		11	3		
1592	3/11/19 9:02	March		26	4		5
482	3/11/19 9:03	March		31	3		
1429	3/11/19 9:17	March		33	3		

② = 5 ③ = 22 ④ = 9 ⑤ = 0 = 36 total

1430	3/11/19 9:18	March		13	4		
1593	3/11/19 10:23	March		15	3		
426	3/11/19 10:24	March		40	4		10
427	3/11/19 10:44	March		21	3		
1431	3/11/19 10:51	March		20	4		
671	3/11/19 11:07	March		22	3		
428	3/11/19 11:15	March		21	3		
483	3/11/19 11:32	March		21	3		15
1594	3/11/19 12:00	March		25	3		
1432	3/11/19 12:07	March		29	3		
484	3/11/19 12:17	March		73	4		
1595	3/11/19 12:20	March		102	3		
1433	3/11/19 12:21	March		86	3		20
~~1434~~	~~3/11/19 12:26~~	~~March~~		16	2		
584	3/11/19 12:49	March		29	2		
672	3/11/19 12:57	March		33	3		
585	3/11/19 13:05	March		63	2		
586	3/11/19 13:46	March		23	4		25
1596	3/11/19 14:04	March		35	3		
1597	3/11/19 14:33	March		40	3		
1435	3/11/19 14:44	March		94	2		
485	3/11/19 15:22	March		16	3		
~~1598~~	~~3/11/19 15:43~~	~~March~~		64	3		30
486	3/11/19 15:50	March		16	4		
~~587~~	~~3/11/19 15:53~~	~~March~~		25	3		
588	3/11/19 16:27	March		26	2		
673	3/11/19 16:47	March		44	4		
487	3/11/19 17:14	March		9	3		35
589	3/11/19 17:22	March		28	4		③⑥
488	[illegible]						

Bad! →

March 11, 2019 Surge Event Scenario

Pts 1 → 5

weighted

11:07	# level	3 → 3
11:15		3 → 3
11:32		3 → 3
12:00		3 → 3 (12)
12:07		3 → 3
1:00 hr		(15) → 1/5.A

Pts 6 → 10

12:17	4 → 2
12:20	3 → 3
12:21	3 → 3
12:26	2 → 4 (12) ≈ "24"
12:49	2 → 4
0:32	(16) ["32"] → S.A.

15" in 3/4 hr

~"22.5" assumed #

Pts 11 → 15 (16)

12:57	3 → 3
13:05	2 → 4
13:46	4 → 2
14:04	3 → 3
14:33	3 → 3
1:36	(15)

Pt 16 (14:44 2 → 4) (19)

(31)/2hr = (15½)

35/2.5 hr =

Exhibit C3

(Selected Data For Analysis Based on Surge Event Criteria)

3. Data of May 20, 2019

MAY 20 2019 (1) = 0 (2) = 6 (3) = 13 (4) = 14 (5) 34 [redacted]

554	5/20/19 6:00	May	22	4	
365	5/20/19 7:09	May	44	3	
1553	5/20/19 7:42	May	18	3	
366	5/20/19 8:06	May	18	4	
383	5/20/19 8:27	May	21	2	5
1097	5/20/19 8:31	May	76	4	
384	5/20/19 8:38	May	22	2	
1554	5/20/19 8:43	May	63	4	
~~1555~~	~~5/20/19 8:47~~	~~May~~	60	4	
385	5/20/19 8:51	May	25	2	10
367	5/20/19 9:09	May	11	3	
555	5/20/19 9:15	May	106	4	
1098	5/20/19 9:17	May	61	4	
1099	5/20/19 9:35	May	1	2	
164	5/20/19 9:42	May	13	3	15
368	5/20/19 9:47	May	8	3	
556	5/20/19 10:03	May	58	4	
165	5/20/19 10:12	May	56	3	
166	5/20/19 10:42	May	62	5	
~~386~~	~~5/20/19 10:47~~	~~May~~	17	2	20
1556	5/20/19 10:58	May	56	3	
167	5/20/19 11:29	May	63	4	
168	5/20/19 11:32	May	66	4	
1101	5/20/19 11:49	May	49	3	
387	5/20/19 11:50	May	48	3	25
1100	5/20/19 11:53	May	18	3	
388	5/20/19 12:03	May	71	4	
1102	5/20/19 12:11	May	104	3	
645	5/20/19 12:33	May	159	3	
646	5/20/19 12:34	May	81	4	30
[illegible]	5/20/19 13:12	May	[illegible]	2	
[illegible]	[illegible]	May	[illegible]	3	
[illegible]	[illegible]	May	[illegible]	2	
[illegible]	[illegible]	May	62	1	34
[illegible]	[illegible]	May	[illegible]	8	

Exhibit C4

(Selected Data For Analysis Based on Surge Event Criteria)

4. Data of June 2, 2019 (CONTRASTING Data)

June 2
2019

1 (0) 2. (2) 3. (16) 4. (12) 5. (0) - 30

1971	6/2/19 2:09	June		186	2			
1972	6/2/19 6:44	June		25	3			
1503	6/2/19 7:02	June		23	3			ML 0900
1350	6/2/19 7:40	June		12	4			
1973	6/2/19 9:26	June		15	3			5
1351	6/2/19 9:56	June		28	3			
1353	6/2/19 10:12	June		27	3		[redacted]	
1352	6/2/19 10:13	June		7	4		[redacted]	
1354	6/2/19 10:18	June		20	4		[redacted]	
1504	6/2/19 10:28	June		23	4		[redacted]	10
1892	6/2/19 10:34	June		26	3		[redacted]	
1893	6/2/19 10:44	June		33	3		[redacted]	
1355	6/2/19 11:01	June		30	3		[redacted]	
1356	6/2/19 11:03	June		32	4		[redacted]	15
1891	6/2/19 11:16	June		24	3		[redacted]	
1894	6/2/19 11:36	June		25	3		[redacted]	
1975	6/2/19 11:39	June		39	4			
1290	6/2/19 11:45	June		33	4			
1357	6/2/19 13:05	June		65	2			
1974	6/2/19 13:09	June		21	3		20	ML
1976	6/2/19 14:01	June		29	4			
1895	6/2/19 14:20	June		26	3			
1358	6/2/19 14:34	June		14	3			
1291	6/2/19 14:43	June		14	4			
1746	6/2/19 15:44	June		22	4		25	
1977	6/2/19 16:12	June		11	4			
1359	6/2/19 16:50	June		7	3			
1505	6/2/19 17:01	June		12	4			
1978	6/2/19 17:34	June		10	3			
1360	6/2/19 18:14	June		15	3		30	
