# Supplementary material for: Cumulative Weighted-Average Acuity Level: A Novel Clinician-Level Metric to Detect Emergency Department Surge Conditions in Real Time
Source: West J Emerg Med. 2026 Oct 13;27(6):1516–24. doi: 10.5811/westjem.66241 (PMC13761938; doi:10.5811/westjem.66241)
Supplement: Supplementary file 2 [file wjem-27-1516-s001.docx]

**Supplementary Appendix A**

**Combinatorial Feasibility Tables and Generalized Mathematical Formalization**

CWAAL: A Novel Clinician-Level Metric for Real-Time Emergency Department Surge Detection

Kenneth Berry, MD

**PART I: COMBINATORIAL FEASIBILITY TABLES**

**Overview and Rationale**

The CWAAL feasibility threshold of ≤12 (±1) for a single emergency physician was derived by systematic enumeration of all clinically plausible patient acuity combinations for Levels 2, 3, and 4 — the acuity levels most frequently encountered during observed surge events at both study sites. Each combination was evaluated against single-physician capacity within a one-hour window using the heuristic time-demand framework described in the manuscript Methods section (weight × 10 minutes per patient).

Two separate enumerations were performed: (1) all 27 three-patient combinations relevant to Hospital B, where surges typically involved three simultaneous high-acuity patients; and (2) all 81 four-patient combinations relevant to Hospital A, where surges typically involved four simultaneous high-acuity patients. The feasibility threshold was established from the Hospital A four-patient analysis, where CWAAL = 12 represents the critical inflection point between feasible and not-feasible single-physician management. The Hospital B three-patient analysis confirmed that all 27 possible combinations are feasible (≤12), which is consistent with the threshold derived from Hospital A.

A clinically important implication of these tables is that CWAAL = 12 is not a midpoint but an upper bound: it is the maximum CWAAL value at which every possible combination of three-to-four high-acuity patients can theoretically be managed within a 60-minute window. The 19 four-patient combinations that produce exactly CWAAL = 12 represent approximately 120 minutes of aggregate care demand (12 × 10 minutes), which precisely equals two physician-hours — the absolute upper limit of feasibility within a single 60-minute window.

**Table A1: Combination Distribution Summaries**

The following tables summarize the frequency distribution of CWAAL values across all enumerated combinations for Hospital B (three-patient) and Hospital A (four-patient) scenarios. Full combination listings follow in Tables A2 and A3.

| **Table A1a — Hospital B: Three-Patient Combination Distribution (n=27)** | | | | | | | | |
| --- | --- | --- | --- | --- | --- | --- | --- | --- |
| **CWAAL 6** | **CWAAL 7** | **CWAAL 8** | **CWAAL 9** | **CWAAL 10** | **CWAAL 11** | **CWAAL 12** |  |  |
| 1 | 3 | 6 | 7 | 6 | 3 | 1 |  |  |
| All 27 Hospital B combinations are feasible (≤12). Distribution is symmetric around CWAAL=9. Peak at CWAAL=9 (7 combinations). Range: 6–12. | | | | | | | | |
| **Table A1b — Hospital A: Four-Patient Combination Distribution (n=81)** | | | | | | | | |
| **CWAAL 8** | **CWAAL 9** | **CWAAL 10** | **CWAAL 11** | **CWAAL 12** | **CWAAL 13** | **CWAAL 14** | **CWAAL 15** | **CWAAL 16** |
| 1 | 4 | 10 | 16 | 19 | 16 | 10 | 4 | 1 |
| 50 of 81 combinations feasible (≤12); 31 not feasible (≥13). Distribution symmetric around CWAAL=12. Peak at CWAAL=12 (19 combinations). Range: 8–16. The inflection at CWAAL=12 establishes the single-physician feasibility threshold: this is the highest CWAAL value at which all combinations remain within the 60-minute physician-hour, and simultaneously the most common combination (19/81, 23.5%). | | | | | | | | |

**Table A2: Hospital B — All 27 Three-Patient Combinations**

Each row represents one possible combination of three patients arriving simultaneously, drawn from Acuity Levels 2 (Emergent, weight 4), 3 (Urgent, weight 3), and 4 (Non-urgent, weight 2). Combinations are listed in lexicographic order. All 27 combinations are feasible (CWAAL ≤12), confirming that the threshold is not binding for three-patient surge scenarios under single-physician coverage.

| **#** | **Patient 1 Level** | **Patient 2 Level** | **Patient 3 Level** | **Weight 1** | **Weight 2** | **Weight 3** | **CWAAL Sum** | **Time Demand (min)** | **Feasible (<=12)** |
| --- | --- | --- | --- | --- | --- | --- | --- | --- | --- |
| 1 | 2 | 2 | 2 | 4 | 4 | 4 | **12** | ~120 | YES |
| 2 | 2 | 2 | 3 | 4 | 4 | 3 | 11 | ~110 | YES |
| 3 | 2 | 2 | 4 | 4 | 4 | 2 | 10 | ~100 | YES |
| 4 | 2 | 3 | 2 | 4 | 3 | 4 | 11 | ~110 | YES |
| 5 | 2 | 3 | 3 | 4 | 3 | 3 | 10 | ~100 | YES |
| 6 | 2 | 3 | 4 | 4 | 3 | 2 | 9 | ~90 | YES |
| 7 | 2 | 4 | 2 | 4 | 2 | 4 | 10 | ~100 | YES |
| 8 | 2 | 4 | 3 | 4 | 2 | 3 | 9 | ~90 | YES |
| 9 | 2 | 4 | 4 | 4 | 2 | 2 | 8 | ~80 | YES |
| 10 | 3 | 2 | 2 | 3 | 4 | 4 | 11 | ~110 | YES |
| 11 | 3 | 2 | 3 | 3 | 4 | 3 | 10 | ~100 | YES |
| 12 | 3 | 2 | 4 | 3 | 4 | 2 | 9 | ~90 | YES |
| 13 | 3 | 3 | 2 | 3 | 3 | 4 | 10 | ~100 | YES |
| 14 | 3 | 3 | 3 | 3 | 3 | 3 | 9 | ~90 | YES |
| 15 | 3 | 3 | 4 | 3 | 3 | 2 | 8 | ~80 | YES |
| 16 | 3 | 4 | 2 | 3 | 2 | 4 | 9 | ~90 | YES |
| 17 | 3 | 4 | 3 | 3 | 2 | 3 | 8 | ~80 | YES |
| 18 | 3 | 4 | 4 | 3 | 2 | 2 | 7 | ~70 | YES |
| 19 | 4 | 2 | 2 | 2 | 4 | 4 | 10 | ~100 | YES |
| 20 | 4 | 2 | 3 | 2 | 4 | 3 | 9 | ~90 | YES |
| 21 | 4 | 2 | 4 | 2 | 4 | 2 | 8 | ~80 | YES |
| 22 | 4 | 3 | 2 | 2 | 3 | 4 | 9 | ~90 | YES |
| 23 | 4 | 3 | 3 | 2 | 3 | 3 | 8 | ~80 | YES |
| 24 | 4 | 3 | 4 | 2 | 3 | 2 | 7 | ~70 | YES |
| 25 | 4 | 4 | 2 | 2 | 2 | 4 | 8 | ~80 | YES |
| 26 | 4 | 4 | 3 | 2 | 2 | 3 | 7 | ~70 | YES |
| 27 | 4 | 4 | 4 | 2 | 2 | 2 | 6 | ~60 | YES |
| All 27 three-patient combinations of Acuity Levels 2, 3, and 4 are feasible (CWAAL ≤12). The feasibility threshold therefore derived from the Hospital A four-patient analysis (Table A2), not from Hospital B data. Blue = feasible; amber = boundary (CWAAL=12); Time Demand = CWAAL × 10 minutes (heuristic). Level 2 = Emergent (weight 4); Level 3 = Urgent (weight 3); Level 4 = Non-urgent (weight 2). | | | | | | | | | |

**Table A3: Hospital A — All 81 Four-Patient Combinations**

Each row represents one possible combination of four patients arriving simultaneously, drawn from Acuity Levels 2, 3, and 4. Feasible combinations (CWAAL ≤12) are listed first, followed by not-feasible combinations (CWAAL ≥13). Amber shading indicates the 19 boundary combinations at exactly CWAAL = 12.

| **FEASIBLE COMBINATIONS (CWAAL ≤12) — 50 of 81** | | | | | | | |
| --- | --- | --- | --- | --- | --- | --- | --- |
| **#** | **Patient 1 Level** | **Patient 2 Level** | **Patient 3 Level** | **Patient 4 Level** | **CWAAL Sum** | **Time Demand (min)** | **Feasible (≤12)** |
| 9 | 2 | 2 | 4 | 4 | **12** | ~120 | YES |
| 15 | 2 | 3 | 3 | 4 | **12** | ~120 | YES |
| 17 | 2 | 3 | 4 | 3 | **12** | ~120 | YES |
| 18 | 2 | 3 | 4 | 4 | 11 | ~110 | YES |
| 21 | 2 | 4 | 2 | 4 | **12** | ~120 | YES |
| 23 | 2 | 4 | 3 | 3 | **12** | ~120 | YES |
| 24 | 2 | 4 | 3 | 4 | 11 | ~110 | YES |
| 25 | 2 | 4 | 4 | 2 | **12** | ~120 | YES |
| 26 | 2 | 4 | 4 | 3 | 11 | ~110 | YES |
| 27 | 2 | 4 | 4 | 4 | 10 | ~100 | YES |
| 33 | 3 | 2 | 3 | 4 | **12** | ~120 | YES |
| 35 | 3 | 2 | 4 | 3 | **12** | ~120 | YES |
| 36 | 3 | 2 | 4 | 4 | 11 | ~110 | YES |
| 39 | 3 | 3 | 2 | 4 | **12** | ~120 | YES |
| 41 | 3 | 3 | 3 | 3 | **12** | ~120 | YES |
| 42 | 3 | 3 | 3 | 4 | 11 | ~110 | YES |
| 43 | 3 | 3 | 4 | 2 | **12** | ~120 | YES |
| 44 | 3 | 3 | 4 | 3 | 11 | ~110 | YES |
| 45 | 3 | 3 | 4 | 4 | 10 | ~100 | YES |
| 47 | 3 | 4 | 2 | 3 | **12** | ~120 | YES |
| 48 | 3 | 4 | 2 | 4 | 11 | ~110 | YES |
| 49 | 3 | 4 | 3 | 2 | **12** | ~120 | YES |
| 50 | 3 | 4 | 3 | 3 | 11 | ~110 | YES |
| 51 | 3 | 4 | 3 | 4 | 10 | ~100 | YES |
| 52 | 3 | 4 | 4 | 2 | 11 | ~110 | YES |
| 53 | 3 | 4 | 4 | 3 | 10 | ~100 | YES |
| 54 | 3 | 4 | 4 | 4 | 9 | ~90 | YES |
| 57 | 4 | 2 | 2 | 4 | **12** | ~120 | YES |
| 59 | 4 | 2 | 3 | 3 | **12** | ~120 | YES |
| 60 | 4 | 2 | 3 | 4 | 11 | ~110 | YES |
| 61 | 4 | 2 | 4 | 2 | **12** | ~120 | YES |
| 62 | 4 | 2 | 4 | 3 | 11 | ~110 | YES |
| 63 | 4 | 2 | 4 | 4 | 10 | ~100 | YES |
| 65 | 4 | 3 | 2 | 3 | **12** | ~120 | YES |
| 66 | 4 | 3 | 2 | 4 | 11 | ~110 | YES |
| 67 | 4 | 3 | 3 | 2 | **12** | ~120 | YES |
| 68 | 4 | 3 | 3 | 3 | 11 | ~110 | YES |
| 69 | 4 | 3 | 3 | 4 | 10 | ~100 | YES |
| 70 | 4 | 3 | 4 | 2 | 11 | ~110 | YES |
| 71 | 4 | 3 | 4 | 3 | 10 | ~100 | YES |
| 72 | 4 | 3 | 4 | 4 | 9 | ~90 | YES |
| 73 | 4 | 4 | 2 | 2 | **12** | ~120 | YES |
| 74 | 4 | 4 | 2 | 3 | 11 | ~110 | YES |
| 75 | 4 | 4 | 2 | 4 | 10 | ~100 | YES |
| 76 | 4 | 4 | 3 | 2 | 11 | ~110 | YES |
| 77 | 4 | 4 | 3 | 3 | 10 | ~100 | YES |
| 78 | 4 | 4 | 3 | 4 | 9 | ~90 | YES |
| 79 | 4 | 4 | 4 | 2 | 10 | ~100 | YES |
| 80 | 4 | 4 | 4 | 3 | 9 | ~90 | YES |
| 81 | 4 | 4 | 4 | 4 | 8 | ~80 | YES |
| **NOT FEASIBLE (CWAAL ≥13) — 31 of 81** | | | | | | | |
| **#** | **Patient 1 Level** | **Patient 2 Level** | **Patient 3 Level** | **Patient 4 Level** | **CWAAL Sum** | **Time Demand (min)** | **Feasible (≤12)** |
| 1 | 2 | 2 | 2 | 2 | **16** | ~160 | NO |
| 2 | 2 | 2 | 2 | 3 | **15** | ~150 | NO |
| 3 | 2 | 2 | 2 | 4 | **14** | ~140 | NO |
| 4 | 2 | 2 | 3 | 2 | **15** | ~150 | NO |
| 5 | 2 | 2 | 3 | 3 | **14** | ~140 | NO |
| 6 | 2 | 2 | 3 | 4 | **13** | ~130 | NO |
| 7 | 2 | 2 | 4 | 2 | **14** | ~140 | NO |
| 8 | 2 | 2 | 4 | 3 | **13** | ~130 | NO |
| 10 | 2 | 3 | 2 | 2 | **15** | ~150 | NO |
| 11 | 2 | 3 | 2 | 3 | **14** | ~140 | NO |
| 12 | 2 | 3 | 2 | 4 | **13** | ~130 | NO |
| 13 | 2 | 3 | 3 | 2 | **14** | ~140 | NO |
| 14 | 2 | 3 | 3 | 3 | **13** | ~130 | NO |
| 16 | 2 | 3 | 4 | 2 | **13** | ~130 | NO |
| 19 | 2 | 4 | 2 | 2 | **14** | ~140 | NO |
| 20 | 2 | 4 | 2 | 3 | **13** | ~130 | NO |
| 22 | 2 | 4 | 3 | 2 | **13** | ~130 | NO |
| 28 | 3 | 2 | 2 | 2 | **15** | ~150 | NO |
| 29 | 3 | 2 | 2 | 3 | **14** | ~140 | NO |
| 30 | 3 | 2 | 2 | 4 | **13** | ~130 | NO |
| 31 | 3 | 2 | 3 | 2 | **14** | ~140 | NO |
| 32 | 3 | 2 | 3 | 3 | **13** | ~130 | NO |
| 34 | 3 | 2 | 4 | 2 | **13** | ~130 | NO |
| 37 | 3 | 3 | 2 | 2 | **14** | ~140 | NO |
| 38 | 3 | 3 | 2 | 3 | **13** | ~130 | NO |
| 40 | 3 | 3 | 3 | 2 | **13** | ~130 | NO |
| 46 | 3 | 4 | 2 | 2 | **13** | ~130 | NO |
| 55 | 4 | 2 | 2 | 2 | **14** | ~140 | NO |
| 56 | 4 | 2 | 2 | 3 | **13** | ~130 | NO |
| 58 | 4 | 2 | 3 | 2 | **13** | ~130 | NO |
| 64 | 4 | 3 | 2 | 2 | **13** | ~130 | NO |
| 50 of 81 four-patient combinations are feasible (≤12); 31 are not feasible (≥13). The critical inflection point is at CWAAL = 12: combinations ≤11 are feasible within a single 60-minute physician-hour; combinations ≥13 exceed single-physician capacity. Amber shading = boundary combinations (CWAAL = 12); blue = feasible; red = not feasible. Level 2 = Emergent (weight 4); Level 3 = Urgent (weight 3); Level 4 = Non-urgent (weight 2). Time Demand = CWAAL × 10 minutes (heuristic estimate; pending formal time-motion validation). Combinations are listed in lexicographic order. | | | | | | | |

**PART II: GENERALIZED MATHEMATICAL FORMALIZATION**

This section presents a generalized formalization of the CWAAL framework, extending the single-physician, single-zone model described in the manuscript to multi-zone, multi-clinician emergency department configurations. The formalization is provided for methodological transparency and to facilitate adaptation of the framework to diverse ED settings. All parameters remain empirical estimates subject to prospective calibration.

**Section II.1: Variable Definitions**

| **Symbol** | **Variable Name** | **Definition and Notes** |
| --- | --- | --- |
| **Cᵢⱼ(t)** | **CWAAL for clinician i in zone j at time t** | Cumulative weighted acuity sum; the core metric computed in real time |
| **n(t)** | **Number of active patients at time t** | Patients under active management within the defined time window |
| **aₖ** | **Acuity level of patient k** | Integer 1–5 per the five-level triage classification (Level 1 = critical, Level 5 = minor) |
| **wₖ** | **Weight of patient k** | Inverted acuity weight: Level 1=5, Level 2=4, Level 3=3, Level 4=2, Level 5=1 |
| **θₖ** | **Partial completion factor for patient k** | 1.0 for full management; 0.5x for patients partially managed prior to surge interval (heuristic) |
| **Θᵢ** | **Capacity threshold for clinician i** | <=12 (±1) for single ED physician; <=18 (±2) for physician-APC team |
| **α** | **APC capacity coefficient** | 0.8 physician-equivalents (empirical estimate; requires prospective calibration) |
| **Z** | **Alert function (Boolean)** | Z=1 (surge alarm) if Cᵢⱼ(t) > Θᵢ; Z=0 (no alarm) otherwise |
| **T_w** | **Time window** | 60-minute rolling window used to define the active patient cohort for CWAAL calculation |
| **d_k** | **Estimated time demand for patient k** | wₖ × 10 minutes (heuristic base unit; pending formal time-motion validation) |

**Section II.2: Governing Equations**

**Equation 1: Real-Time Rolling Weighted Load (Single Clinician, Single Zone)**

**Cᴵⱼ(t) = Σₖ [ wₖ × θₖ ] for all k ∈ active patients of clinician i in zone j at time t**

Where the sum is taken over all n(t) patients currently under active management by clinician i within zone j at time t. Patients partially managed prior to the defined surge interval receive a reduced weight (θₖ = 0.5×) to account for work already completed; all other patients receive full weight (θₖ = 1.0×). The 0.5× discount is a heuristic estimate and should be empirically calibrated in future work.

**Equation 2: Zone-Level Capacity Threshold**

**Θᴵ = Θ_phys + (α × n_APP)**

Where Θ_phys = 12 (±1) is the single-physician feasibility threshold, α = 0.8 is the APC capacity coefficient (empirical estimate), and n_APP is the number of APPs actively supporting clinician i. For a single physician with one APC: Θᴵ = 12 + (0.8 × 1) = 12.8, conventionally rounded to ≤18 (±2) to reflect the uncertainty in the α estimate. For a physician without APC coverage: Θᴵ = 12 (±1). The APC coefficient α = 0.8 was derived retrospectively from the Hospital B DDT data and requires prospective calibration.

**Equation 3: Boolean Surge Alert Function**

**Zᴵⱼ(t) = 1	if Cᴵⱼ(t) > Θᴵ	(surge alarm active)**

**Zᴵⱼ(t) = 0	if Cᴵⱼ(t) ≤ Θᴵ	(no alarm)**

The alert function produces a binary output: 1 (alarm) when the clinician's current CWAAL exceeds the capacity threshold, and 0 (no alarm) otherwise. In practice, a tiered alert system is proposed (watch at CWAAL ≥10, alert at CWAAL ≥12, alarm at CWAAL ≥18), as described in Table 4 of the manuscript. The binary formulation above represents the alarm tier.

**Equation 4: Multi-Zone Extension**

**Z_ED(t) = max{ Zᴵⱼ(t) }	for all i clinicians, j zones**

For multi-zone EDs with multiple clinicians, the department-level surge alert is the maximum alert state across all clinician-zone pairs. This ensures that a surge condition in any zone triggers the department-level response, regardless of conditions in other zones. Each clinician's CWAAL is computed independently using Equation 1, and each clinician's threshold Θᴵ reflects their specific staffing configuration using Equation 2.

**Section II.3: Worked Example — Single Physician, Surge Onset 11:00**

The following example illustrates real-time CWAAL calculation for a single emergency physician at surge onset, demonstrating the threshold transition from watch to alert to alarm as additional high-acuity patients arrive.

Setting: Hospital B, morning shift. Physician has two pre-existing patients (Levels 3 and 4) partially managed before 11:00 (assigned 0.5× weight). Four new high-acuity patients arrive between 11:00 and 11:30.

| **Patient** | **Acuity Level** | **Weight (wₖ)** | **Prior Mgmt? (θₖ)** | **Adjusted Weight** | **Running CWAAL** | **Threshold (Θ)** | **Status** |
| --- | --- | --- | --- | --- | --- | --- | --- |
| 1 (pre-existing) | 3 | 3 | 0.5x | 1.5 | 1.5 | <=12 | **Watch** |
| 2 (pre-existing) | 4 | 2 | 0.5x | 1.0 | 2.5 | <=12 | **Watch** |
| 3 (new arrival) | 2 | 4 | 1.0x | 4.0 | 6.5 | <=12 | **Watch** |
| 4 (new arrival) | 3 | 3 | 1.0x | 3.0 | 9.5 | <=12 | **Alert** |
| 5 (new arrival) | 2 | 4 | 1.0x | 4.0 | 13.5 | <=12 | **ALARM** |
| 6 (new arrival) | 3 | 3 | 1.0x | 3.0 | 16.5 | <=12 | **ALARM** |
| Worked example: Single ED physician, surge onset 11:00. Patients 1-2 were pre-existing (0.5x weight). Patients 3-6 arrive during surge window (1.0x weight). CWAAL crosses the alert threshold (>12) with Patient 5, triggering the alarm state. Supplemental staffing should be requested at this point. θk = partial completion factor; CWAAL = cumulative sum of adjusted weights. | | | | | | | |

In this example, the surge alarm threshold (Θ = 12) is crossed with the arrival of Patient 5. The appropriate response per the proposed tiered protocol is immediate activation of supplemental staffing. If an APC were present, the effective threshold would be ≤18, and the alarm would not trigger until a CWAAL of 18 is exceeded, providing additional operational margin.

**Section II.4: Implementation Notes and Limitations**

The mathematical formalization presented here is intended as a transparent specification of the CWAAL framework, not a validated clinical protocol. Several parameters require empirical calibration before operational deployment:

1. The 10-minute base time unit (weight × 10 min) is a heuristic derived from clinical experience. Formal time-motion studies are needed to calibrate per-acuity-level time demands across physician types, institutions, and patient populations.
2. The partial completion discount (θₖ = 0.5×) is a heuristic. Time-and-motion data could support a continuous discount function based on elapsed care time.
3. The APC capacity coefficient (α = 0.8) was derived retrospectively from a small dataset (n=8 surge events). Prospective studies with larger samples are needed to establish a robust estimate.
4. The threshold values (≤12 for single physician, ≤18 for physician-APC team) were derived from and evaluated on the same dataset. They should be treated as preliminary working hypotheses, not validated standards.
5. The framework currently measures physician acuity burden only. Extensions incorporating nursing capacity, bed availability, and ancillary service response times would improve completeness but require additional modeling work.

Implementation in standard EMR systems (Meditech, Epic, Cerner) would require only triage acuity data and patient assignment data — both of which are captured in real time during routine clinical workflow. Automated CWAAL calculation and display within the ED tracking board is technically feasible without additional data infrastructure, and could enable prospective threshold validation studies at scale.

Supplementary Appendix A — CWAAL Manuscript — Kenneth Berry MD — 2026
